# Loss of Material, Personal, and Social Resources and Their Impact on Mental Health Among Young People Not in Employment, Education, or Training

**DOI:** 10.1002/jad.70024

**Published:** 2025-08-10

**Authors:** Xintai Chen, Randolph C. H. Chan

**Affiliations:** ^1^ Department of Social Work The Chinese University of Hong Kong Shatin Hong Kong

**Keywords:** mental health, NEETs, optimism, perceived economic status, perceived social acceptance

## Abstract

**Introduction:**

Young people face heightened employment pressure, particularly those in the school‐to‐work transition phase. Although earlier studies have indicated that young people who are not in employment, education, or training (NEET) experience considerable psychological distress, limited attention has been paid to understanding why they are at risk of poor mental health. Grounded in the conservation of resources theory, this study examined the mental health differences between NEETs and those who are in employment, education, or training (non‐NEET). We also investigated how loss of resources could explain the differences.

**Methods:**

Based on the Chinese Family Panel Studies dataset in 2020, the study's sample included 5336 individuals aged from 16 to 35 in China (46% females; M_age_ = 28.13, SD_age_ = 4.99). Approximately 14.25% of the participants were considered NEETs.

**Results:**

The results revealed that NEETs showed significantly higher levels of depression symptoms and lower levels of subjective well‐being than non‐NEETs. Path analysis revealed that, compared to non‐NEETs, NEETs reported significantly lower levels of key resources, including perceived economic status, sense of optimism, and perceived social acceptance. These diminished resources mediated the relationship between NEET status and adverse mental health outcomes, specifically elevated depression symptoms and reduced subjective well‐being.

**Conclusions:**

This study highlights that NEETs experience poorer mental health than non‐NEETs, not only due to material deprivation, but as a result of personal and social resource loss. Therefore, beyond providing financial assistance, it is essential to develop evidence‐based social services that focus on restoring NEETs’ sense of optimism and rebuilding social acceptance.

## 1 Introduction

School‐to‐work transition is a key developmental task for young people (Schoon and Heckhausen 2019). However, unemployment and underemployment are prevalent among young people in many regions, which has become a global concern (United Nations 2020). In China, the unemployment rate of young people (aged from 16 to 24) increased to 21.3% in June 2023, which was significantly higher than the overall unemployment rate of 5.2% (National Bureau of Statistics of China 2025). A recent systematic review pointed out that multiple dimensions of risk factors contribute to this outcome (Rahmani and Groot 2023). Faced with challenging conditions, such as economic recession (Schoon and Bynner 2019), reduced labor demand following the pandemic (Liang et al. 2022), and persistent gender stereotypes in the labor market (Hoff et al. 2024), young people often exhibit hesitancy when transitioning into the workforce. Meanwhile, deciding on a career is not easy for young people (Udayar et al. 2020), especially for those with lower self‐efficacy and limited human capital (Guay et al. 2006; Ng et al. 2023). These environmental and individual factors hinder some students from successfully transitioning from school to work, rendering them at high risk of becoming disengaged or even fully become not in employment, education, or training (NEET) (Ngai et al. 2024).

NEET no longer signifies merely an abstract status; it has come to denote the individuals who embody and experience that status (Caroleo et al. 2020). NEETs is a term to describe young people not in employment, education, or training (Mascherini 2019), which encompasses a heterogeneous group of young people (Russell 2014). They can be disaggregated into seven subgroups, including “re‐entrant, short‐term unemployed, long‐term unemployed, unavailable because of illness or disability, unavailable because of family responsibilities, discouraged workers, and other inactive” (Mascherini 2019). Regardless of the specific category they fall into, reintegrating these young people into the labor market is an urgent agenda. Previous research has indicated that their mental health problems may continue to influence their later employment and life (Huegaerts et al. 2020; Lee et al. 2019). Although previous research has documented the impact of educational and employment disruption on mental health (Gariépy et al. 2022), a comprehensive framework explaining the underlying causes remains scarce. This study addresses that gap by applying the conservation of resources (COR) theory to examine mental health disparities between NEETs and their non‐NEET counterparts. Specifically, we investigate which types of resource loss (material, personal, and social resources) contribute to these disparities.

To our knowledge, few studies have applied the COR theory to the NEET population, particularly using nationally representative data from China. The timing of data collection in 2020 is also significant. During this period, the labor market became increasingly uncertain, with intensified competition among job seekers (MacGowan et al. 2021). Chinese young people began to collectively reflect on the pressures of hyper‐competition, a phenomenon widely discussed through the discourse of “involution” (nei juan) (Zhou et al. 2022). This reflection gave rise to the “lying flat” (tang ping) movement, in which some young people opted out of the relentless pursuit of success (Lu et al. 2023; Zheng et al. 2023). Our data captures mental health disparities just before this societal shift, providing a unique snapshot of youth well‐being during a time of growing uncertainty.

### 1.1 Mental Health Disparities Between NEETs and Non‐NEETs

The World Health Organization (2022) emphasized that mental health is a multifaceted construct that encompasses not only the absence of mental disorders and psychosocial disability but also the presence of mental well‐being. According to a recent meta‐analysis, being NEET may amplify the risk of mental health issues for young people (Gariépy et al. 2022). A cross‐national research from the European Union showed that NEETs, excluding unpaid care workers, were significantly more likely to report lower levels of psychological well‐being than employed peers (Jongbloed and Giret 2022). Furthermore, Basta et al. (2019) found that older and long‐term NEETs were at higher risk for experiencing symptoms of depression and anxiety.

The mental health disparities between NEETs and non‐NEETs may be explained through the conservation of resources theory. The theory posits that individuals experience stress when their resources are threatened or lost (Hobfoll 1989; Hobfoll et al. 2018). Employment, for example, not only provides material resources but also supports identity formation, social status, and interpersonal connections (Jahoda 1981). Becoming NEET often entails the loss of these opportunities, which can lead to psychological distress. While prior research has linked NEET status to poor health and well‐being (Klug et al. 2019), few studies have explored which specific resources are most affected. This study, therefore, investigated the mental health differences between NEETs and non‐NEETs and attempted to examine how resource loss contributes to these differences.

### 1.2 Loss of Resources Among NEETs

Resources are defined as valued entities that individuals strive to obtain, retain, and protect (Hobfoll et al. 2018). Hobfoll (1989) categorized resources into four types: object resources, personal resources, condition resources, and energy resources. However, this broad definition has drawn criticism for being overly inclusive (Halbesleben et al. 2014). To address this limitation, ten Brummelhuis and Bakker (2012) proposed a more refined typology of resources. They introduced a two‐dimensional framework based on source (ranging from contextual to personal) and transience (ranging from structural to volatile), which helps clarify the nature of different resources. Their model also incorporated social support as a key component of individual resources. In contrast, Hobfoll (1989, 2018) excluded social support from his core resource categories due to its dual potential to be both beneficial and burdensome. Instead, perceived social acceptance, such as close interpersonal relationships, was classified under condition resources. Building on this conceptual foundation, Zwiebach et al. (2010) operationalized a threefold resource taxonomy encompassing material, personal, and social resources (Zwiebach et al. 2010). The present study adopts this framework to investigate the resource loss experienced by NEETs.

Material resources refer to tangible assets such as income, savings, and financial stability (Hobfoll 2010). NEETs, by virtue of being disengaged from the labor market, are more likely to experience economic hardship and downward mobility (Oliver et al. 2014; Ralston et al. 2022). This can lead to a cycle of poverty and prolonged detachment from education or employment (Fabrizi and Rocca 2024; Mussida and Sciulli 2023; Oliver et al. 2014). NEETs may therefore experience threats to essential living resources, which, according to the COR theory, can provoke psychological distress. This is supported by systematic reviews that have consistently shown lower socioeconomic status to be a strong predictor of poor mental health outcomes (Ritterman 2007; Rivenbark et al. 2019).

Personal resources include traits and skills such as self‐efficacy, self‐esteem, and optimism (Hobfoll 1989, 2010). Given the setbacks experienced in the labor market, along with underdeveloped educational attainment and limited work experience (Bynner and Parsons 2002; Rahmani and Groot 2023), NEETs may have greater uncertainty about their future prospects (Rodrigues et al. 2024). These experiences can erode self‐efficacy (Mortimer et al. 2016) and diminish optimism, which is an often overlooked but critical personal resource. Given the increasingly competitive labor market in China (Liu et al. 2024), NEETs may face heightened challenges due to their disengagement from further education and skills development (Léné 2011). As a result, they are less likely to feel optimistic about their future. According to the COR theory, a lack of personal resources, such as optimism, can increase vulnerability to poor mental health outcomes. Previous studies have identified these resources as protective factors for NEETs and unemployed people. For instance, optimism has been shown to buffer the negative impact of unemployment on mental health (Lai and Wong 1998) and to serve as a predictor of mental health (Conversano et al. 2010; Lee 2008; Rincón Uribe et al. 2022).

Social resources refer to the support, information, and opportunities individuals can access through their social relationships. Specifically, they involve perceived social acceptance derived from positive and supportive relationships (Brock et al. 1998). However, due to the stigma associated with unemployment, NEETs may experience feelings of shame regarding their current status (Peterie et al. 2019). These feelings can lead to self‐seclusion (Su and Wong 2022) and gradual social withdrawal from the external environment (Eckhard 2022). In Chinese culture, the concept of “face” is a deeply rooted value, representing an individual's pursuit of respect and recognition within their social group (Xiaoying Qi 2011), and is often tied to the honor of the entire family (Hu 1944). As a result, the pressure to maintain “face” can further limit NEETs' opportunities to cultivate social acceptance and may further exacerbate the emotional distance between parents and themselves. Research has shown that Chinese parents often have specific goals and expectations for their children, including employment and educational attainment (To et al. 2021). This suggests that parent–child relationships may be conditional, with parental approval and support dependent on the child meeting these expectations. For NEETs who are unable to fulfill such expectations, forming close and supportive family relationships can become increasingly difficult, thereby experiencing less social acceptance. Perceived social acceptance is a crucial determinant of mental health (Romani et al. 2021; Witvliet et al. 2010; Zimmer–Gembeck et al. 2007). Without access to this social resource, NEETs may be at greater risk of experiencing poor mental health outcomes.

### 1.3 Aims of the Present Research

Situated within the Chinese context, this study aimed to (1) examine demographic differences between NEETs and non‐NEETs, (2) determine differences in depression symptoms and subjective well‐being between NEETs and non‐NEETs, and (3) investigate the underlying mechanisms contributing to poorer mental health among NEETs based on the conservation of resources theory. Three hypotheses were proposed as follows:

Hypothesis 1 NEETs would show higher levels of depression symptoms and lower levels of subjective well‐being than non‐NEETs.

Hypothesis 2 NEETs would exhibit lower levels of material, personal, and social resources than non‐NEETs.

Hypothesis 3 The mental health differences between NEETs and non‐NEETs would be mediated by loss of material, personal, and social resources.

## 2 Methods

### 2.1 Study Design

The data of this study are drawn from the 2020 wave of the China Family Panel Studies (CFPS). CFPS is a nationwide survey covering twenty‐five provinces in China. Households were recruited using multistage probability proportional to size (PPS) sampling with implicit stratification (Xie and Hu 2014). In the 2020 wave of the CFPS, data collection was primarily conducted through telephone interviews due to the COVID‐19 pandemic. Information was gathered from a total of 28,530 participants. The survey covered a wide range of topics, including family structure, interpersonal relationships, economic conditions, significant life events, health status, behaviors, and mental well‐being.

### 2.2 Participants

In line with youth policy issued by the State Council of the People's Republic of China (2017), individuals aged 14 to 35 are classified as young people. However, according to the Labour Law of the People's Republic of China (1995), the minimum legal working age is 16. Therefore, for the analysis in this paper, the age of young people is from 16 to 35. Based on this criterion, a total of 8366 participants from the 2020 CFPS dataset met the initial eligibility requirements. To ensure data completeness, only participants who provided responses to all variables included in the analysis were retained. This resulted in a final sample of 5887 participants, with no missing values on the analyzed measures.

CFPS provided the “School” variable to identify the schooling status of participants. We classify those who were identified “at school” or “on vocation” status into non‐NEETs. “Employ” variable was also generated by CFPS to check the current employment status of individuals. Those who were “employed” were identified as non‐NEETs in this study. Participants who were not in employment were asked the question, “Why are you currently unemployed?” They were provided with the following choices, including options such as “not needing/wanting to work,” “engaging in housework,” “too old and physically feeble (mainly referring to elderly farmers)”, “having a disability/illness that prevents working,” “not being able to find a suitable job,” “being affected by COVID‐19,” and an “other reasons” category. Participants who chose these options were identified as NEETs. On the other hand, individuals who chose “attending school or training” were identified as non‐NEET. Based on the responses to these questions, 839 individuals were classified as NEETs, accounting for 14.25% of young people in this dataset, whereas 5048 individuals were classified as non‐NEETs.

“Engaging in housework” was the predominant reason that young people have been NEETs, accounting for 65.67% of NEETs (*n* = 551). Approximately 20.98% of young people (*n* = 176) were classified as NEETs due to reasons such as “not needing/wanting to work,” “not finding a suitable job,” or “the influence of COVID‐19.” Meanwhile, 2.38% of young people (*n* = 20) attributed their unemployment to disability or illness, while 10.97% of NEETs (*n* = 92) chose “other reasons.”

Although being responsible for doing the housework is a reason to be NEETs, this particular group of participants was excluded from the analysis due to the involvement of complex factors related to domestic work patterns (Bianchi et al. 2012), gender equality (Samtleben and Müller 2022), and the perception of housework as unpaid work (Wang and Lu 2023). After excluding the 551 caregivers, the analysis focused on the remaining participants (*N* = 5336), among whom 288 individuals were classified as NEETs.

### 2.3 Measures

#### 2.3.1 Material, Personal, and Social Resources

Items were chosen from the CFPS, which were the most closely aligned with the definition of these three resources. Material resources were operationalized as the perceived economic status, reflecting their subjective evaluation of the adequacy of their overall material conditions. In CFPS, participants were asked to respond: “What is your relative income level in your local area?”, which was rated on a 5‐point Likert scale from 1 (very low) to 5 (very high). Personal resources were operationalized as a sense of optimism, which captured individuals' belief in positive future outcomes, independent of external circumstances (Gavrilov‐Jerković et al. 2014). In the CFPS, this was measured by the item: “How confident are you about your future?”, which closely resembles an item from the Personal Optimism Scale (Gavrilov‐Jerković et al. 2014). Participants responded on a 5‐point Likert scale from 1 (strongly disagree) to 5 (strongly agree), reflecting their level of optimism. Social resources were assessed through perceived social acceptance, measured by the item: “Do you think you are popular?” This item closely resembles one from the Perceived Social Acceptance Scale (Schmidt et al. 2015). The item was rated on a 5‐point Likert scale from 1 (lowest) to 5 (highest).

#### 2.3.2 Depression Symptoms

The abbreviated eight‐item version Center for Epidemiologic Studies Depression Scale was used to measure depression symptoms in the past week (e.g., “I felt that everything I did was an effort.”) This scale has been demonstrated to be reliable and valid for assessing depression symptoms (Karim et al. 2015). Items were rated on a four‐point Likert scale from 1 (never: less than 1 day) to 4 (most of the time: 5–7 days). The Cronbach's *α* of the scale in this study was 0.76.

#### 2.3.3 Subjective Well‐Being

Participants were asked to assess their level of subjective well‐being by responding to the question, “To what extent do you think you are happy?” using an 11‐point Likert scale from 0 (lowest) to 10 (highest).

### 2.4 Data Analysis

Descriptive statistics were used to characterize the demographics and mental health (depression symptoms and subjective well‐being). Chi‐square tests were used to compare demographic differences between NEETs and non‐NEETs. Independent‐sample *t*‐tests were conducted to explore differences between NEETs and non‐NEETs in resources (i.e., perceived economic status, sense of optimism, and perceived social acceptance), depression symptoms, and subjective well‐being. Correlation was used to determine the relationships between resources and mental health.

Path analysis was conducted to examine whether perceived economic status, sense of optimism, and perceived social acceptance would mediate the association of NEET status with mental health, after controlling for demographics (i.e., gender, age, education, household living area, and yearly family income level). The mean of CESD‐8 was calculated and used as an indicator of depression symptoms. Mediation analysis was conducted to examine the pathways linking NEET status, resources, and mental health. The 95% confidence intervals were estimated by the Bootstrap method with 1000 resamples (Wen and Liu 2020). SPSS version 28.0 and Amos 28 were used to conduct analyses.

## 3 Results

### 3.1 Demographic Characteristics of NEETs

Chi‐square test results revealed significant differences between NEETs and non‐NEETs in terms of gender, age, and family income (see Table 1). Specifically, a significantly higher proportion of NEETs were female (55.2%) compared to non‐NEETs (45.5%), while males were more prevalent among non‐NEETs (54.5%) than NEETs (44.8%) (*χ*² = 10.42, *p* < 0.01). Age distribution also varied significantly, with NEETs more likely to be aged 16–24 (39.2%) compared to non‐NEETs (23.3%), while a smaller proportion of NEETs were aged 25–35 (60.8%) relative to non‐NEETs (76.7%) (χ² = 37.67, *p* < 0.001). In terms of income, NEETs (RMB¥ 35150 or below: 23.6%, RMB¥ 35,151–60,000: 19.8%, RMB¥ 60,001–90,000: 20.1%, RMB¥ 90,001–144,000: 21.5%, RMB¥ 144,001 or above: 14.9%) were more likely to come from lower‐income households compared to non‐NEETs (RMB¥ 35,150 or below: 9.3%, RMB¥ 35,151–60,000: 18.3%, RMB¥ 60,001–90,000: 19.1%, RMB¥ 90,001–144,000: 25%, RMB¥ 144,001 or above: 28.4%) (*χ*² = 74.85, *p* < 0.001).

**Table 1:** Demographic characteristics of the participants.

	Analyzed sample (*N* = 5336)	NEETs (*n* = 288)	Non‐NEETs (*n* = 5048)	Group difference
	*n* (%)/Mean (SD)	*n* (%)/Mean (SD)	*n* (%)/Mean (SD)	*χ* ^2^/*t*‐value
Gender				10.42**
Male	2882 (54.0%)	129 (44.8%)	2753 (54.5%)	
Female	2454 (46.0%)	159 (55.2%)	2295 (45.5%)	
Age group				37.67***
16–24	1290 (24.2%)	113 (39.2%)	1177 (23.3%)	
25–35	4046 (75.8%)	175 (60.8%)	3871 (76.7%)	
Household living area				2.48
Urban	3018 (56.6%)	150 (52.1%)	2868 (56.8%)	
Rural	2318 (43.4%)	138 (47.9%)	2180 (43.2%)	
Education Level				4.91
Secondary or below	522 (9.8%)	39 (13.5%)	483 (9.6%)	
Postsecondary	2813 (52.7%)	144 (50.0%)	2669 (52.9%)	
Bachelor or above	2001 (37.5%)	105 (36.5%)	1896 (37.6%)	
Yearly family income				74.85***
RMB¥ 35,150 or below	538 (10.1%)	68 (23.6%)	470 (9.3%)	
RMB¥ 35,151–60,000	981 (18.4%)	57 (19.8%)	924 (18.3%)	
RMB¥ 60,001–90,000	1020 (19.1%)	58 (20.1%)	962 (19.1%)	
RMB¥ 90,001–144,000	1322 (24.8%)	62 (21.5%)	1260 (25.0%)	
RMB¥ 144,001 or above	1475 (27.6%)	43 (14.9%)	1432 (28.4%)	
Mental health				
Depression symptoms	1.68 (0.46)	1.74 (0.51)	1.67 (0.46)	2.19*
Subjective well‐being	7.48 (1.92)	7.2 (2.18)	7.50 (1.90)	−2.16*
Resources				
Perceived economic status	2.80 (0.90)	2.56 (1.05)	2.81 (0.89)	−4.11***
Sense of optimism	4.14 (0.84)	3.98 (0.91)	4.15 (0.83)	−3.38**
Perceived social acceptance	6.89 (1.71)	6.54 (1.98)	6.91 (1.69)	−3.11**

* *p* < 0.05

** *p* < 0.01

*** *p* < 0.001.

### 3.2 Mental Health and Loss of Resources

The results showed that depression symptoms, subjective well‐being, perceived economic status, sense of optimism, and perceived social acceptance were significantly different between NEETs and non‐NEETs (see Table 1). Specifically, NEETs demonstrated significantly higher levels of depression symptoms (*t* = 2.19, *p* < 0.05) and lower levels of subjective well‐being (*t* = −2.16, *p* < 0.05) than non‐NEETs, which supports Hypothesis 1. The results also indicated that NEETs had significantly lower perceived economic status (*t* = −4.11, *p* < 0.001), sense of optimism (*t* = −3.38, *p* < 0.01), and perceived social acceptance (*t* = −3.11, *p* < 0.01) than non‐NEETs. The findings provide support for hypothesis 2.

Pearson's product‐moment correlation coefficient was estimated for the relationships between the perceived economic status, sense of optimism, perceived social acceptance, and mental health (see Table 2). Depression symptoms were negatively correlated with perceived economic status (*r* = −0.12, *p* < 0.001), sense of optimism (*r* = −0.28, *p* < 0.001), and perceived social acceptance (*r* = −0.19, *p* < 0.001). Meanwhile, subjective well‐being was positively correlated with perceived economic status (*r* = 0.22, *p* < 0.001), sense of optimism (*r* = 0.37, *p* < 0.001), and perceived social acceptance (*r* = 0.43, *p* < 0.001).

**Table 2:** Descriptive statistics and intercorrelations of the variables (*N* = 5336).

	1	2	3	4	5
1. Perceived economic status	—				
2. Sense of optimism	0.25***	—			
3. Perceived social acceptance	0.18***	0.26***	—		
4. Depression symptoms	−0.12***	−0.28***	−0.19***	—	
5. Subjective well‐being	0.22***	0.37***	0.43***	−0.35***	—

* *p* < 0.05

** *p* < 0.01

*** *p* < 0.001.

### 3.3 A Mediation Model of Mental Health Between NEETs and Non‐NEETs

**Figure 1:**
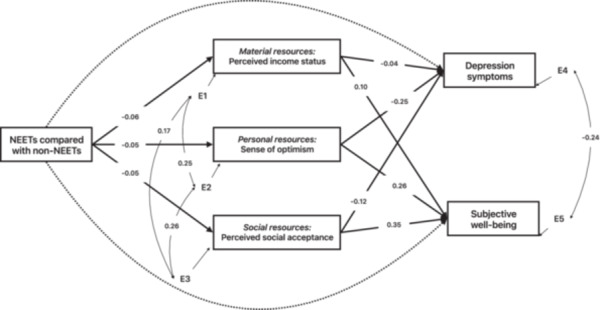
Underlying mechanisms contributing to poorer mental health among NEETs compared to non‐NEETs. Controlling for gender, age, education, household living area, and family yearly income level; solid lines indicate statistically significant paths, and dotted lines denote nonsignificant paths.

Figure 1 presents the mediation model of the effect of NEET status on depression symptoms and subjective well‐being, mediated by perceived economic status, sense of optimism, and perceived social acceptance. The model showed an acceptable model fit: *χ* = 164.90 (*df* = 27, *p* < 0.001), CFI = 0.99, NFI = 0.99, IFI = 0.99, TLI = 0.97, RMSEA = 0.03, SRMR = 0.02. NEET status was negatively related to perceived economic status (*β* = −0.06, *p* < 0.001), sense of optimism (*β* = −0.05, *p* < 0.001), and perceived social acceptance (*β* = −0.05, *p* < 0.001). Perceived economic status was negatively associated with depression symptoms (*β* = −0.04, *p* < 0.01) and positively related to subjective well‐being (*β* = 0.10, *p* < 0.001). Sense of optimism was negatively associated with depression symptoms (*β* = −0.25, *p* < 0.001) and positively related to subjective well‐being (*β* = 0.26, *p* < 0.001). Perceived social acceptance was negatively associated with depression symptoms (*β* = −0.12, *p* < 0.001) and positively related to subjective well‐being (*β* = 0.35, *p* < 0.001). Table 3 presents the unstandardized and standardized path coefficients for the hypothesized model.

**Table 3:** Unstandardized and standardized parameter estimates for the hypothesized model.

	Unstandardized	Standardized
	*B* (SE)	*β*
NEETs → Perceived income status	−0.26 (0.05)***	−0.06***
NEETs → Sense of optimism	−0.17 (0.05)***	−0.05***
NEETs → Perceived social acceptance	−0.37 (0.10)***	−0.05***
NEETs → Depression symptoms	0.02 (0.03)	0.01
NEETs → Subjective well‐being	−0.003 (0.1)	0.000
Perceived income status → Depression symptoms	−0.02 (0.01)***	−0.04**
Perceived income status → Subjective well‐being	0.21 (0.03)***	0.10***
Sense of optimism → Depression symptoms	−0.14 (0.01)***	−0.25***
Sense of optimism → Subjective well‐being	0.61 (0.03)***	0.26***
Perceived social acceptance → Depression symptoms	−0.03 (0.004)***	−0.12***
Perceived social acceptance → Subjective well‐being	0.39 (0.01)***	0.35***
Perceived income status ↔ Sense of optimism	0.19 (0.01)***	0.25***
Perceived income status ↔ Perceived social acceptance	0.27 (0.02)***	0.17***
Sense of optimism ↔ Perceived social acceptance	0.37 (0.02)***	0.26***
Depression symptoms ↔ Subjective well‐being	−0.17 (0.01)***	−0.24***

* *p* < 0.05

** *p* < 0.01

*** *p* < 0.001.

The indirect effects of NEET status on depression symptoms and subjective well‐being were examined using a bootstrapping analysis. Consistent with the hypothesis 3, the results revealed that significant indirect effects were observed for NEETs on depression symptoms via the perceived economic status (*β* = 0.01, 95% CI = 0.001, 0.01), sense of optimism (*β* = 0.02, 95% CI = 0.01, 0.04), and perceived social acceptance (*β* = 0.01, 95% CI = 0.004, 0.02). In addition, perceived economic status (*β* = −0.05, 95% CI = −0.09, −0.03), sense of optimism (*β* = −0.104 95% CI = −0.17, −0.04), and perceived social acceptance (*β* = −0.15, 95% CI = −0.24, −0.05) significantly mediated the association of NEET status with subjective well‐being.

## 4 Discussion

Based on nationally representative data from China, this study provides empirical evidence of significant mental health disparities between NEETs and non‐NEETs, while also exploring the underlying mechanisms that may account for these differences. Prior research has identified heightened vulnerability to NEET status among females (Odoardi et al. 2023) and individuals from low‐income families (Noh and Lee 2017; Rahmani and Groot 2023). Consistent with these findings, our results showed that the proportion of females and individuals from relatively lower‐income households was significantly higher among NEETs compared to non‐NEETs. Additionally, the data revealed an overrepresentation of individuals aged 16–24 within the NEET group, suggesting that younger people in China faced particular challenges in accessing employment or continuing education during 2020. These findings align with the concerns about the youth unemployment crisis, which was exacerbated by the COVID‐19 pandemic (Blustein et al. 2020; Churchill 2021; Ganson et al. 2021).

The results demonstrate notable mental health disparities between NEETs and non‐NEETs. Specifically, NEETs reported significantly higher levels of depression symptoms and lower levels of subjective well‐being than their non‐NEET counterparts, aligning with findings from previous research (Basta et al. 2019; Gutiérrez‐García et al. 2017; Klug et al. 2019). However, few studies have explored the underlying mechanisms of these disparities. Originally developed to explain stress through the lens of resource loss (Hobfoll 1989), COR theory has been widely applied in occupational burnout research (Chu and Chou 2024; Prapanjaroensin et al. 2017). In this study, we extend COR theory to highlight how resource depletion contributes to mental health outcomes among NEETs. This theoretical lens allows us to shift the focus from perceived personal deficits to resource loss, thereby contributing to efforts to reduce stigma surrounding the NEET population.

In response to critiques regarding the broad and sometimes ambiguous definition of resources in COR theory (Halbesleben et al. 2014), we adopt the threefold resource taxonomy proposed by Zwiebach et al. (2010), which categorizes resources into material, personal, and social domains. Drawing on this framework, and supported by our findings, we propose that the loss of these resources, operationalized as perceived economic status (material), optimism (personal), and perceived social acceptance (social), may independently account for the mental health differences observed between NEETs and non‐NEETs.

Our findings indicate that perceived economic status serves as a mediator in explaining the mental health differences between NEETs and non‐NEETs. Previous studies have shown that poverty is both a risk factor and a consequence of being NEET (Mussida and Sciulli 2023; Noh and Lee 2017), and our findings lend further support to the latter. As suggested by previous studies (Ritterman 2007; Rivenbark et al. 2019), NEETs tend to perceive their economic status as lower than that of non‐NEETs due to the absence of personal income sources. In addition, our analysis shows that a greater proportion of NEETs come from relatively low‐income families, which leaves them suffering from increased economic constraints. These financial pressures may, in turn, contribute to heightened depression symptoms and reduced levels of subjective well‐being.

The loss of personal resources, particularly optimism, also helps explain the mental health disparities between NEETs and non‐NEETs. Consistent with previous research (Goldman‐Mellor et al. 2016), our findings indicate that NEETs report significantly lower levels of optimism than their non‐NEET counterparts. This pattern should be interpreted within the broader sociocultural context of involution, a hypercompetitive atmosphere experienced by young people in China. The intensifying labor market competition exacerbates the divide between NEETs and non‐NEETs, leaving NEETs with fewer opportunities and limited prospects due to lower educational attainment and insufficient work experience (Léné 2011). In response to these constraints, some NEETs may proactively lower their future aspirations to avoid competition and repeated failure (Tong 2023). This coping mechanism is reflected in the growing appeal of the “lying flat” lifestyle, which rejects conventional success markers such as stable employment and upward mobility (Song et al. 2024; Wang and Wang 2021). Increasingly, some young people have come to accept precarious employment and adopt a more passive lifestyle characterized by minimal ambition (Han and Lu 2023). This mirrors the hikikomori phenomenon in Japan (Uchida and Norasakkunkit 2015), where reduced optimism is associated with disengagement from future‐oriented goals.

The “lying flat” movement gained widespread resonance among Chinese young people in 2021 and has been considered a psychological compensation strategy for coping with life stress (Wang et al. 2024; Zheng et al. 2023). However, empirical evidence suggests that this strategy may not yield positive mental health outcomes. For instance, Lu et al. (2024) found that lying flat was negatively associated with mental health, indicating that many young people may struggle to genuinely embrace reduced aspirations. Rather than representing a deliberate rejection of societal norms, lying flat may reflect a deeper erosion of optimism. Our findings support this interpretation, indicating that NEETs in 2020 were particularly vulnerable to optimism depletion under the prevailing hypercompetitive climate. Within the framework of COR theory, optimism is regarded as a key personal resource that can mobilize or activate other resources (Hobfoll 2010; ten Brummelhuis and Bakker 2012). When deprived of optimism, young people are more likely to experience depression symptoms and reduced subjective well‐being. Prior studies have shown that unemployed individuals with higher levels of optimism tend to report lower psychological distress (Achdut and Refaeli 2020) and greater life satisfaction (Duffy et al. 2013). Consistent with these findings, our study demonstrates that diminished optimism among NEETs is significantly associated with increased depression symptoms and lower levels of subjective well‐being.

Lack of perceived social acceptance may also contribute to the mental health disparities between NEETs and non‐NEETs. As suggested by previous studies (Su and Wong 2022), NEETs may tend to isolate themselves and become socially withdrawn after disengaging from education or employment. Additionally, the stigma and prejudice surrounding unemployment may further weaken their relationships with family, peers, and the broader community (Miller et al. 2015). In the Chinese cultural context, where “face” is a significant concern, parents may experience anxiety about their children's NEET status and unintentionally transfer this anxiety to their children. The combination of these factors may contribute to a reduction in social resources among NEETs and exacerbate their already challenging mental health conditions.

### 4.1 Practical Implications

Our findings provide evidence of the detrimental impact of disrupted career development on NEETs' material, personal, and social resources, which collectively contribute to poorer mental health outcomes. These results underscore the need for support services targeting NEETs to adopt a holistic, resource‐oriented approach that addresses their mental health concerns. Drawing on the resource caravan principle from COR theory (Hobfoll et al. 2018), we emphasize that resources can be cultivated and sustained through supportive social environments. While state welfare programs, such as financial support, can provide essential material resources for NEETs, it is important to consider the accessibility, equity, and the risk of fostering dependency (McPherson 2021). In contrast, expanding employment opportunities for young people offers a more stable and empowering form of material support (Schoon and Bynner 2019). To address the pressure of an increasingly competitive labor market, policymakers should move beyond restrictive hiring regulations (e.g., banning age or gender requirements) and instead promote better employment‐person fit. This shift would help young people find roles that align with their strengths and aspirations, rather than forcing conformity to rigid criteria.

Furthermore, our findings highlight the importance of rebuilding personal and social resources. Many NEETs experience diminished optimism and social acceptance, particularly when navigating a highly competitive environment. To address this, interventions should focus on fostering positive future expectations and guiding young people toward diverse career pathways that reflect their unique strengths and potential employability. Family support also plays a critical role in this process. Encouraging parents to offer unconditional support, rather than linking approval to achievements, can help NEETs strengthen familial relationships and their social acceptance. Finally, it is essential to prioritize targeted interventions for the vulnerable subgroups identified in this study, particularly females, individuals aged 16 to 24, and those from low‐income families. These groups face heightened risks and are likely to benefit from support strategies that are specifically tailored to address their unique challenges and needs.

### 4.2 Limitations

Despite the theoretical and practical contribution of this study, several limitations should be considered. First, we used single‐item measures rather than well‐validated scales to assess optimism and perceived social acceptance, such as the Personal Optimism Scale (Gavrilov‐Jerković et al. 2014) and the Perceived Social Acceptance Scale (Brock et al. 1998). This approach may have oversimplified the measurement of these constructs. Second, although national data were utilized to present a general overview of the NEET conditions among Chinese young people, the cross‐sectional design limits our ability to draw causal inferences. As suggested by Gariépy et al. (2022) in their meta‐analysis, the relationship between NEET status and mental health remains complex and potentially bidirectional. According to COR theory, individuals with fewer resources are less able to acquire new resources, which may lead to a downward spiral of resource loss (Hobfoll 2010). Longitudinal studies are therefore needed to explore the directionality of the relationship between NEET status and mental health, and to examine whether resource loss contributes to prolonged NEET status. In addition, the sequence and rate at which material, personal, and social resources are depleted warrant further investigation. A deeper understanding of these dynamics would enable the timely implementation of targeted strategies to ensure support is delivered when NEETs are most vulnerable. Third, the concept of “involution” and “lying flat” among young people, while discussed in this study, remains largely hypothetical and lacks substantial empirical evidence. Future research should investigate the potential interaction between resource loss, “involution,” and the “lying flat” mindset among young people in China.

### 4.3 Conclusions

The career developmental trajectories of young people have been significantly disrupted by the challenges and uncertainties brought about by the COVID‐19 pandemic. This study uses national data to investigate the mental health conditions and disparities between NEETs and non‐NEETs in China. Our findings support the hypothesis that NEETs are at greater risk of losing material, personal, and social resources, which in turn contributes to poorer mental health outcomes. Given the heightened mental health risks associated with prolonged exposure NEET status, it is crucial to implement evidence‐based interventions that help young people regain these critical resources and create supportive environments that enable them to re‐engage with education, employment, and society.

## Ethics Statement

The China Family Panel Studies (CFPS) were reviewed and approved by the Institutional Review Board of Peking University.

## Conflicts of Interest

The authors declare no conflicts of interest.

## Data Availability

The data are derived from the China Family Panel Studies (CFPS), a project supported by the 985 Program of Peking University and conducted by the Institute of Social Science Survey at Peking University. The data are openly available at https://www.isss.pku.edu.cn/cfps/en/data/public/index.htm.
